# Molecular, Biochemical, and Clinical Characterization of Thirteen Patients with Glycogen Storage Disease 1a in Malaysia

**DOI:** 10.1155/2022/5870092

**Published:** 2022-09-13

**Authors:** Siti Aishah Abdul Wahab, Yusnita Yakob, Mohd Khairul Nizam Mohd Khalid, Noraishah Ali, Huey Yin Leong, Lock Hock Ngu

**Affiliations:** ^1^Molecular Diagnostics Unit, Specialised Diagnostic Centre, Institute for Medical Research, National Institute of Health, Ministry of Health, Jalan Pahang 50586, Kuala Lumpur, Malaysia; ^2^IEM & Genetic Unit, Nutrition Metabolic and Cardiovascular Research Centre, Institute for Medical Research, National Institute of Health, Ministry of Health, Kuala Lumpur, Malaysia; ^3^Department of Genetics, Hospital Kuala Lumpur, Ministry of Health, Kuala Lumpur, Malaysia

## Abstract

**Background:**

Glycogen storage disease type 1a (GSD1a) is a rare autosomal recessive metabolic disorder characterized by hypoglycaemia, growth retardation, lactic acidosis, hepatomegaly, hyperlipidemia, and nephromegaly. GSD1a is caused by a mutation in the *G6PC* gene encoding glucose-6-phosphatase (G6Pase); an enzyme that catalyses the hydrolysis of glucose-6-phosphate (G6P) to phosphate and glucose.

**Objective:**

To elaborate on the clinical findings, biochemical data, molecular genetic analysis, and short-term prognosis of 13 GSD1a patients in Malaysia.

**Methods:**

The information about 13 clinically classified GSD1a patients was retrospectively studied. The *G6PC* mutation analysis was performed by PCR-DNA sequencing.

**Results:**

Patients were presented with hepatomegaly (92%), hypoglycaemia (38%), poor weight gain (23%), and short stature (15%). Mutation analysis revealed nine heterozygous mutations; eight previously reported mutations (c.155 A > T, c.209 G > A, c.226 A > T, c.248 G > A, c.648 G > T, c.706 T > A, c.1022 T > A, c.262delG) and a novel mutation (c.325 T > C). The most common mutation found in Malaysian patients was c.648 G > T in ten patients (77%) of mostly Malay ethnicity, followed by c.248 G > A in 4 patients of Chinese ethnicity (30%). A novel missense mutation (c.325 T > C) was predicted to be disease-causing by various *in silico* software.

**Conclusions:**

The establishment of *G6PC* molecular genetic testing will enable the detection of presymptomatic patients, assisting in genetic counselling while avoiding the invasive methods of liver biopsy.

## 1. Introduction

**Figure 1:**
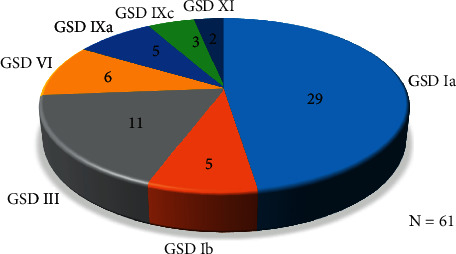
Number of patients diagnosed at the National Referral Centre, Genetic Clinic, Hospital Kuala Lumpur, Malaysia, from 1998–2021.

Glycogen storage diseases (GSD) are a group of metabolic disorders of glycogen metabolism. GSD mostly affects the liver, skeletal muscles, heart, and sometimes the central nervous system [1]. There are more than 12 different types, and they are classified based on the deficient enzymes and affected tissues [2]. The most common type is GSD type 1a, representing about 80% of GSD1 patients. GSD1a was first described by Von Gierke in 1929. It is a recessively inherited metabolic disorder with a prevalence of one in 100,000 live births [3]. There are limited prevalence data for GSD in the Malaysian population; however, available data from the national referral centre at Genetic Clinic Hospital Kuala Lumpur from 1998–2021, suggests that the majority of patients have GSD1a (47.6%) as well (Figure 1).

GSD1a (MIM #232220) is caused by the deficiency of glucose-6-phosphatase (G6Pase), an enzyme which catalyses the hydrolysis of glucose-6-phosphate (G6P) to phosphate and glucose. Deficiency of G6Pase causes an increase of G6P in the cytoplasm and triggers alternative metabolic pathways, thus leading to the accumulation of glycogen in glucose-generating organs, including the liver, kidney, and small intestine. G6Pase is a hydrophobic protein located in the endoplasmic reticulum containing 357 amino acids and nine transmembrane helix structures with its NH- and COOH- termini facing the ER lumen and cell cytoplasm, respectively. This enzyme is encoded by *G6PC* gene (OMIM #613742) located on chromosome 17q21, spanning about 12.5kb region and contains 5 exons [4].

The initial diagnosis of GSD1a is based on the clinical presentation and biochemical analysis such as hepatomegaly, hypoglycaemia, lactic acidosis, hypercholesterolemia, hypertriglyceridaemia, and hyperuricaemia which are usually manifested in the infantile period. The confirmation of diagnosis can be made either by measuring the G6Pase activity in liver biopsy tissue or by *G6PC* gene sequencing. Genetic analysis of the *G6PC* gene is preferred as it is less invasive compared to liver biopsy and it also facilitates genetic counselling.

To date, more than 135 unique mutations have been reported in the HGMD (http://www.hgmd.cf.ac.uk/ac/index.php), most of which were missense types, followed by deletion and/or insertion and splicing. In the different ethnic groups, specific mutations were found at high frequencies, such as Arg83Cys in Jewish (98%) and Caucasian (33%), Arg83His in Chinese, and c.648 G > T in East Asians (Japanese 91%, Korean 86.2%, and Chinese 54%) [5–7]. In the present study, we report the clinical, biochemical, molecular analysis, and short-term prognosis of 13 GSD1a patients in Malaysia.

## 2. Materials and Methods

### 2.1. Patient and Sample Collection

Thirteen patients with a clinical diagnosis of GSD 1a who have not had a molecular analysis were referred to the Molecular Diagnostic Unit, Institute of Medical Research for molecular investigation. Patients' medical records were retrospectively reviewed for medical history, clinical examination, and laboratory study results. Standard deviations for height were calculated using standard growth charts from the World Health Organization (WHO) [8]. Routine biochemistry was performed in the clinical laboratory in a Hospital in Kuala Lumpur and the laboratory's reference ranges were provided. Descriptive statistics, including means, minimum, and maximum, were calculated. All statistical analyses were performed using Microsoft Excel. Parents of the affected child were guided to sign the consent form for genetic testing. Approximately 2.5 to 5 ml of peripheral blood in EDTA tubes were taken from patients for molecular genetic analysis of the *G6PC* gene.

### 2.2. Polymerase Chain Reaction

Genomic DNA was extracted using Chemagic Prepito D (Perkin Elmer) and both the quantity and quality of extracted DNA were measured using a NanoDrop ND-1000 Spectrophotometer. Six sets of primers were designed in-house to amplify five coding exons and flanking intronic sequences of the *G6PC* gene including splice sites (Supplement Table 1). PCR was performed in a 50 *µ*l volume containing 50 ng genomic DNA, 0.1 U Taq DNA polymerase, 1X PCR buffer with MgSO_4_, 1 *µ*mol of each primer and 0.2 mM of 10 mM dNTP mix. Amplification was performed using a touchdown PCR protocol as described by [9].

### 2.3. DNA Sequencing and Variant Analysis

Purification of PCR products and Sanger sequencing were performed as described previously [10]. Sequencing results were aligned to the reference sequence of the *G6PC* gene (NM_000151.3) using the SeqScape software v.3.0 (Applied Biosystem) to identify DNA variants. All variants identified were sought in the following database: Human Gene Mutation Database (HGMD) (http://www.hgmd.cf.ac.uk/ac/index.php), Clinvar (https://www.ncbi.nlm.nih.gov/clinvar/), and Genome Aggregation Database (gnomAD) (http://gnomad.broadinstitute.org).

Novel variants were further checked using variant data from 100 genomes of the Singaporean Malays retrieved from the Singapore Sequencing Malay Project (SSMP) (http://phg.nus.edu.sg/StatGen/public_html/SSMP/SSMP_index.html) [11]. Several in silico tools were used to predict the pathogenicity of novel missense mutations by using MutationTaster (http://www.mutationtaster.org) [12], VarSome (https://varsome.com/) [13], and CADD (https://cadd.gs.washington.edu/).

### 2.4. Protein Structure Analysis

The crystal structure for G6Pase is currently not available on the Protein Data Bank (PDB), therefore we used the structure predicted by AlphaFold-2 (AF-2) for this analysis [14]. The PDB file was retrieved from the UniProt database (https://www.uniprot.org/uniprotkb/P35575/entry#structure) and the impact of novel missense mutations on protein structure was predicted by using Missense3D (http://missense3d.bc.ic.ac.uk/∼missense3d/) [15]. Next, we performed *in silico* mutagenesis using FoldX to predict the impact of the novel missense mutation on the thermodynamic stability of the protein structure [16]. The PDB file was first repaired using the FoldX RepairPDB command, and the repaired PDB was subjected to mutagenesis using the BuildModel command. We used the same criteria described by Caswell et al. [17] to interpret the change in free energy of the mutated structure compared to the wild-type structure. Both protein structures (containing native or mutated residues) were visually inspected using PyMOL.

## 3. Results

### 3.1. Clinical Analysis

The clinical features, biochemical and *G6PC* mutations of 13 Malaysian patients from twelve unrelated families with GSD1a are summarized in Table 1. Nine were males (69%) and four were females (31%). The median age of diagnosis was 13 months old (range 9 months to 16 years old). All presented with hepatomegaly except for patient 13 (92%). Other clinical features on presentation were hypoglycaemia (5/13, 38%), poor weight gain (3/13, 23%), and short stature (2/13, 15%). Motor delay, epistaxis, splenomegaly, and gouty arthritis were found in one patient, respectively (8%). Biochemical features included raised hyperlactataemia (mean 4.5 mmol/L, range 1.8–13.4 mmol/L), hypertriglyceridaemia (mean 11 mmol/L, range 5.7–24.6 mmol/L), hyperuricaemia (mean 503 mmol/L, range 228–866 mmol/L), raised alanine aminotransferase (mean 168 U/L, range 29–351 U/L), and raised aspartate aminotransferase (mean 223 U/L, range 87–357 U/L).

Follow-up data (Table 2) were available for 11 patients with a mean age of 9.4 years (median 6 years). Patient 4, who defaulted treatment at 17 years old, died at 20 years old due to sepsis. Dietary advice was frequent complex carbohydrates for all patients, and 9/11 patients (81.8%) took uncooked corn starch with doses ranging from 0.3–1.6 g/kg/feed. No patient used overnight perfusion feed in our cohort. Of the 11 patients with follow-up, seven (63.6%) had short stature, and all (100%) had hyperlactataemia and hypertriglyceridaemia. Seven patients (63.6%) took Allopurinol, an oral xanthine oxidase inhibitor, but despite that, four of them had plasma uric acid above the reference range (>400 *µ*mol/L) at the last follow-up. Two (18.2%) had multiple focal liver lesions on ultrasonography, but none underwent liver biopsy, and serum alpha-fetoprotein was not raised.

### 3.2. G6PC Genotyping

**Figure 2:**
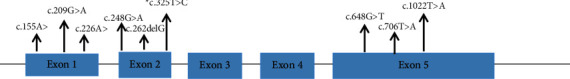
Schematic of nine mutations spanned all exons except exon 3 and 4 were identified in G6PC gene. Mutation c.648 G > T is the most common mutation present in 13 alleles followed with c.248 G > T in four alleles. Novel mutation is labelled with ^*∗*^.

Nine mutations were identified in 13 patients (Table 1). These included five missense mutations (His52Leu, Arg83His, Cys109Arg, Trp236Arg, and Ile341Asn), two nonsense mutations (Trp70^*∗*^ and Lys76^*∗*^), a splice site mutation (c.648 G > T) and a frameshift mutation (c.262delG). Schematic of nine mutations spanned all exons except exon 3 and 4 were identified in G6PC gene are shown at Figure 2. Two recurrent mutations (c.648 G > T and c.Arg83His) were identified in 13 and 4 alleles of the total mutant alleles in our patient cohort, respectively. Carrier status of the parents was confirmed for patients 1, 2, 6, 8, 9, and 10, whereas others were not available for carrier testing. A novel mutation, Cys109Arg, found in this study was not detected in 100 genomes of Singaporean Malay. The novel Cys109Arg was one of the compound heterozygous mutations exhibited in Patients 5 and 12, whereby the c.325 T > C change replaced cysteine with arginine at codon 109 of the G6Pase protein. MutationTaster predicts c.325 T > C to be disease causing and CADD score was 26.9 (deleterious) whereas VarSome classified the variant as uncertain significance/likely pathogenic based on evidence of one moderate (PM2); that is not present in the population database (gnomAD) and two supporting evidence; (PP2) missense variants in G6PC is the common mechanism of the disease and (PP3) 11 pathogenic computational verdict as deleterious effect on the gene.

**Figure 3:**
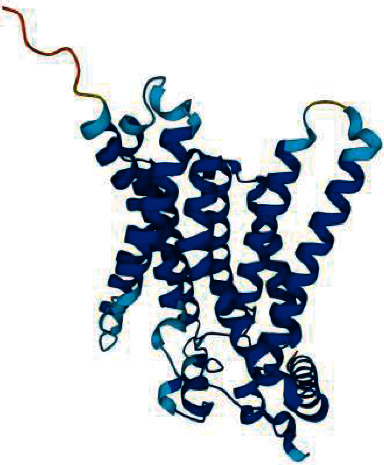
Structure of G6Pase predicted by AlphaFold-2. Dark blue indicates a predicted Local Distance Difference Test (pLDDT) score above 90 and light blue indicates a pLDDT score of between 70 and 90.

The Cys109 residue is located in an extended, unstructured loop region of G6Pase which has a high predicted Local Distance Difference Test (pLDDT) score of 92.99 (Figure 3). This score exceeds the recommended threshold of 70 for the AF-2 model, therefore structural analysis performed using this model is likely to generate reliable predictions [17]. Missense3D predicted that replacement of Cys with Arg would abolish the disulphide bond formed between Cys109 and residue Cys254. This is supported by *in silico* mutagenesis analysis by FoldX that predicted the missense change to severely destabilise the protein structure.

## 4. Discussion

In this paper, we presented the clinical, biochemical, and molecular findings of 13 GSD1a patients from Malaysia. The spectrum of mutations identified in our patients were similar to HGMD, where missense changes were the most common type of mutation in the *G6PC* gene.

The most common mutation in our cohort was c.648 G > T, which was found in ten patients, followed by c.248 G > T that was identified in four patients. The c.648 G > T mutation was common in patients of Asian ancestry as reported in patients from Japanese (91%) [18], South Korean (75%) [21], Chinese ethnicity (54%) [22], and Malay ethnicity (78%) [20]. Our findings supported this observation that the c.648 G > T mutation was prevalent among patients of Malay origin. This variant was shown to alter splicing by producing an aberrant transcript that eliminated 91 nucleotides resulting in an altered reading frame and premature termination Kajihara (1995). The c.248 G > A mutation was identified in four unrelated Chinese patients, which was also in agreement with the high prevalence of this mutation among patients of Chinese origin [20].

Both nonsense mutations (Trp70^*∗*^ and Lys76^*∗*^) and a frameshift mutation (c.262delG) were predicted to create a premature stop codon. A nonsense mutation occurring 11 amino acids from the carboxyl terminus was devoid of enzymatic activity [19] and, because of this, the shorter enzymes produced by the above-mentioned mutations could also result in loss of function. More importantly, Lys76 was one of the active site residues in G6Pase and substitution with asparagine was shown to abolish enzyme activity [23].

The novel mutation c.325 T > C (p.Cys109Arg) has been identified in two different GSD1a patients in the heterozygous state. Furthermore, Cys109Arg is positioned at the luminal loop and is shown to play a crucial role in the catalytic activity of the enzyme. This has been demonstrated by Shieh et al. [24] and Angaroni et al. [25] as the mutation Thr108Ile and Glu110Lys located at the luminal loop were shown to have inactivated *G6Pase* activity. Another possibility as shown by *in silico* analysis is the potential for the missense mutation to destabilise the structure of G6Pase which also could severely impact enzymatic activity. Despite the absence of functional studies for our novel mutation, it was predicted to be pathogenic by *in silico* program and since the mutation was found in patients from ethnic Malay, the absence of mutations in the Singaporean Malay genome database showed that the new allele was extremely rare in Malay population.

To date, there is no clear genotype-phenotype relation for GSD1a, even though several studies have proposed that some mutations may be associated with certain phenotypes. Nevertheless, some studies have reported the relationship between homozygous c.648 G > T with the level of severity in hepatocellular carcinoma [26–28], but we have yet to determine this phenotype in our patients as they are still young. However, two of our homozygous c.648 G > T patients (Patient 8 and 10) showed a severe phenotype, presenting in infancy with hepatomegaly, hypoglycaemia, hyperlactataemia, and hypertriglyceridaemia. Unfortunately, clinical data was incomplete for the third homozygous patient (Patient 11).

The oldest GSD1a patient was diagnosed at 16 years old (patient 13), presenting with gouty arthritis and short stature, demonstrating again that hyperuricaemia in adolescence can be a presenting feature for GSD1a as previously reported [29]. His clinical presentation contrasts with his younger brother (patient (2), who had massive hepatomegaly by the age of 4 years old. There are similar reports of variable phenotypes among affected siblings [29–31], which suggests additional genetic and/or environmental modifying factors [32].

The diagnosis is complicated and challenging because GSD patients exhibit phenotypic heterogeneity. However, gene mutational analysis enables a noninvasive and accurate way of diagnosing type Ia patients. Hence, as prompt and accurate diagnosis is the most important point for the proper treatment of metabolic diseases, next generation sequencing (NGS) can provide the most accurate and cost- and time-efficient approach for the fast diagnosis of the disease as well as overcome the difficulties in analysing diseases with broad clinical and genetic heterogeneity.

## 5. Conclusion

The establishment of a molecular genetic testing service for the *G6PC* gene will allow the diagnosis of GSD1a patients and eliminate the need for a liver biopsy. Besides that, it also enables the detection of presymptomatic patients and assists in genetic counselling. In conclusion, we have characterized both the clinical and molecular aspects of patients with GSD1a in Malaysia. The novel mutation identified in this study will further expand the spectrum of pathogenic mutations associated with GSD1a.

## Figures and Tables

**Table 1:** Clinical features, biochemical and the *G6PC* mutations in GSD1a Malaysian patients.

Pt No	Sex/Ethnicity	Age onset	Age of dx	Initial clinical manifestation	Lactate, mmol/L (ref <2)	Triglyceride, mmol/L (ref <1.7)	Uric acid, *µ*mol/L (ref <400)	ALT/AST, IU/L (ref <42/<51)	Nucleotide changes	Protein changes	Exon	Mutation reported
1#	F/Chinese	7m	9m	Hepatomegaly, hypoglycaemia	5.5	11	687	129/148	c.248 G > A c.1022 T > A	p.Arg83His p.Ile341Asn	2 5	Hwu (1995) Lee (1996)
2^*∗*^#	M/Malay	3y 8 m	3y 8 m	Hepatomegaly	2.6	9.1	569	309/275	c.648 G > T c.706 T > A	p.Leu216 = p.Trp236Arg	5 5	Kajihara [18] Lei [19]
3	M/Chinese	8m	2y 2m	Hepatomegaly, poor weight gain, epistaxis	4.2	24.6	560	351/357	c.262delG c.648 G > T	p.Val88Phefs^*∗*^14 p.Leu216 =	2 5	Lam (1998) Kajihara [18]
4	F/Chinese	ND	6y	Hepatomegaly, short stature, poor weight gain	ND	ND	ND	ND	c.209 G > A c.248 G > A	p.Trp70^*∗*^ p.Arg83His	1 2	Trioche (1999) Hwu (1995)
5	M/Malay	12m	12m	Hepatomegaly, hypoglycaemia	ND	ND	500	ND	c.155 A > T **c.325 T** **>** **C**	p.His52Leu **p.Cys109Arg**	1 2	Rahman [20] **This study**
6#	F/Malay	12m	13m	Hepatomegaly	13.4	11.4	405	89/158	c.226 A > T c.648 G > T	p.Lys76^*∗*^ p.Leu216 =	1 5	Rahman [20] Kajihara [18]
7	M/Chinese	10m	12m	Hepatomegaly, hypoglycaemia	ND	ND	ND	ND	c.248 G > A c.648 G > T	p.Arg83His p.Leu216 =	1 5	Hwu (1995) Kajihara [18]
8#	M/Malay	6m	9m	Hepatomegaly, hypoglycaemia, poor weight gain	5.5	13	451	229/335	Homo c.648 G > T	p.(Leu216 = )	5	Kajihara [18]
9#	M/Chinese	15m	2y 10m	Hepatomegaly	4.2	7.1	269	181/149	c.248 G > A c.648 G > T	p.(Arg83His) p.(Leu216 = )	2 5	Hwu (1995) Kajihara [18]
10#	M/Malay	9m	9m	Hepatomegaly, splenomegaly, motor delay	3.3	6.6	228	156/275	Homo c.648 G > T	p.(Leu216 = )	5	Kajihara [18]
11	M/Malay	ND	4y	Hepatomegaly, hypoglycaemia	ND	ND	ND	ND	Homo c.648 G > T	p.(Leu216 = )	5	Kajihara [18]
12	F/Malay	5m	10m	hepatomegaly	8	11.2	ND	39/87	**c.325 T** **>** **C** c.648 G > T	**p.(Cys109Arg)** p.(Leu216 = )	2 5	**This study** Kajihara [18]
13^*∗*^	M/Malay	13y	16y	Short stature, gouty arthritis	1.8	5.7	866	29/ND	c.648 G > T c.706 T > A	p.Leu216 = p.Trp236Arg	5 5	Kajihara [18] Lei [19]

Abbreviations. ALT, alanine transaminase; AST, aspartate transaminase; Dx, diagnosis; F female; M, male; m, months; ND, not determined; Pt, patient; y, years; ^*∗*^ siblings, #Inherited from parents.

**Table 2:** Follow-up, treatment, and outcome of GSD1a Malaysians patients.

Pt No	Age at last follow-up (yrs)	Short stature (<2SD for age and sex)	Uncooked corn starch (g/kg/feed)	Allopurinol use	Blood lactate, mmol/L (ref <2)	Uric acid, µmol/L (ref <400)	Triglyceride, mmol/L (ref <1.7)	Proteinuria (ref <3.5 mg/mmol creat)	Multiple liver focal lesions (ultrasonography)
1	4	N	1.3	y	6.2	344	5.3	N	N
2	12	Y	0.3	y	3.56	500	15.2	Y	N
3	3	N	1	Y	4.81	531	13.7	N	N
4	17	Y	N	N	17.6	443	8.5	ND	Y
5	ND	ND	ND	ND	ND	ND	ND	ND	ND
6	6	Y	1.6	y	10.9	150	21.5	N	N
7	25	Y	N	Y	3.67	318	8.3	N	Y
8	2	Y	1.3	N	3.1	300	6.4	N	N
9	3	N	0.7	N	4.1	247	5.7	N	N
10	1	N	0.6	N	4.8	279	3.6	N	N
11	ND	ND	ND	ND	ND	ND	ND	ND	ND
12	12	Y	1	Y	6.6	540	8.6	N	N
13	18	Y	0.5	Y	5.2	570	3.3	Y	N

Abbreviations. N, no; ND, not determined; SD, standard deviation; Y, yes.

## Data Availability

The datasets used and analysed during the current study can be obtained from the corresponding author upon reasonable request.

## Abbreviations:

GSD1a: Glycogen storage disease 1a
G6PC: Glucose-6-phosphatase
HGMD: Human Gene Mutation Database.

## Supplementary Materials

Supplementary Materials Table 1 Primers for exon-specific sequencing of G6PC gene.
