# Supplementary material for: Molecular, Biochemical, and Clinical Characterization of Thirteen Patients with Glycogen Storage Disease 1a in Malaysia
Source: Genet Res (Camb). 2022 Sep 13;2022:5870092. doi: 10.1155/2022/5870092 (PMC9489408; doi:10.1155/2022/5870092)
Supplement: Supplementary Materials — Table 1 Primers for exon-specific sequencing of G6PC gene. [file 5870092.f1.docx]

**Supplement**

**Table 1 Primers for exon-specific sequencing of G6PC gene**

| **Exon** | **Forward primer** | **Reverse primer** | **Product length (bp)** |
| --- | --- | --- | --- |
| 1 | TAGCAGAGCAATCACCACCA | TGGGGAAAGCAACTTCTGAT | 396 |
| 2 | GGCAACATGTGAAATCCTTCT | CATGTCCCCTCAAGGTCAGT | 297 |
| 3 | CCCAGATGAGGACCTTTTCA | AGGGGGATGTGAGGAAGAAT | 249 |
| 4 | TAAGTTTGCCAGGCTCCAAC | CTGGAATGCTGGGATTTTGT | 295 |
| 5 | 1. TGTCACCCACTCCTCCAAAC | ATGGGAGCCACTTGCTGA | 400 |
|  | 1. GGGCTGGCTCTCAACTCC | ATACCAGTGCCCATTGCTTC | 346 |
